# A digital Norwegian version of the client satisfaction questionnaire 8: factor validity and internal reliability in outpatient mental health care

**DOI:** 10.1186/s12888-022-04281-8

**Published:** 2022-10-31

**Authors:** Henrik Pedersen, Audun Havnen, Martin Brattmyr, C. Clifford Attkisson, Mariela L. Lara-Cabrera

**Affiliations:** 1 Nidaros Community Mental Health Centre, Division of Psychiatry, St. Olavs University Hospital, Trondheim, Norway; 2 Department of Mental Health, Faculty of Medicine and Health Sciences, Norwegian University of Science and Technology (NTNU), Trondheim, Norway; 3 Department of Psychology, Norwegian University of Science and Technology (NTNU), Trondheim, Norway; 4 Department of Psychiatry, University of California, San Francisco, United States; 5 Tamalpais Matrix Systems, LLC, San Fransisco, United States; 6 Nidelv Community Mental Health Centre, Division of Psychiatry, St. Olavs University Hospital, Trondheim, Norway , Trondheim, Norway

**Keywords:** Patient satisfaction, Community mental health services, Mental disorders, Psychometric properties, Reliability, Validity, Patient-reported experience, Quality of mental healthcare, Quality of care, Satisfaction with treatment

## Abstract

**Background:**

Validated measures of patient-reported experiences are essential for assessing and improving the quality of mental health services and interventions. In Norwegian mental healthcare settings, the Client Satisfaction Questionnaire (CSQ-8) is increasingly being used for this purpose, but the validity and reliability of the Norwegian translation have not been investigated.

**Methods:**

We examined the factor structure and internal consistency of a digitally administrated Norwegian translation of the CSQ-8 in a sample of 338 patients recruited from outpatient treatment. The relationship between satisfaction scores and the change in symptom severity during treatment, measured by the Patient Health Questionnaire-4, was also investigated.

**Results:**

The Norwegian CSQ-8 showed a clear unidimensional structure with one factor explaining 74% of the variance. Internal consistency was very high, with a Cronbach’s alpha of 0.95. Satisfaction showed a small-to-moderate negative relationship with change in symptom severity. Satisfaction scores were negatively skewed, and the presence of ceiling effects is discussed.

**Conclusion:**

Our results support the use of the Norwegian CSQ-8 as a valid and reliable measure of satisfaction with mental healthcare services. Further studies are needed to determine the test-retest reliability of the questionnaire, its sensitivity to change, and to assess its propensity to ceiling effects.

## Background

Over the last several decades, patients’ satisfaction with services has emerged as an essential outcome measure in health care quality assessment and is viewed as a necessary addition to more traditional outcome measures, such as symptom reduction. Patient satisfaction is also a major political issue worldwide, and regarded as an important outcome, with international organizations such as The World Health Organization, The Commonwealth Fund, and the Organisation for Economic Co-operation and Development, emphasizing the importance of conducting both national, and cross-national studies [1]. Larsen et al. [2] have highlighted three main reasons for its importance. First, measuring the clinicians’ perspective alone leads to an incomplete and biased appraisal of services provided. The second reason refers to the need to adhere to legislative mandates of patient involvement. The third reason is the importance of maintaining the interests of vulnerable populations, e.g., people of low socioeconomic status, who may be unable to choose between different health service alternatives.

Although the assessment of patient satisfaction has been stressed as essential for evaluating the quality of mental healthcare [3–6], different systematic reviews have described the patient satisfaction literature as flawed [5, 7, 8]. In particular, the lack of gold standard measures with well-established psychometric properties has been highlighted. In addition, many measures have been developed ad-hoc and used only a handful number of times, which makes it difficult to conduct inter-program comparisons and comparisons across different samples.

The Client Satisfaction Questionnaire (CSQ) is one of the most used questionnaires for measuring patient-reported satisfaction in mental healthcare settings [5, 9]. While different versions exist (varying from 3 to 18 items), the CSQ-8, with eight items,  is the most widely used. Each question is answered on a 4-point scale (possible range from eight to 32, where a higher score indicates a higher level of overall satisfaction with services[2, 10–12].

Globally, the CSQ-8 has been translated into 51 languages [10] and used to assess patient satisfaction in a range of mental healthcare services. The CSQ-8 has been used in studies examining the quality of inpatient forensic services, community and substance abuse services, inpatient services, and outpatient clinical settings[5]. In addition, the CSQ has been used to assess patient satisfaction across various populations, including both voluntary and involuntary admitted patients [13], as well as pre-therapy educational interventions [11, 14], teletherapy [15], and follow-up interventions [16]. Finally, a slightly modified version has been used to evaluate web-based treatments [17].

The original English version of the CSQ-8 showed good internal consistency and one-factor structure [12]. Validated translations include German [18], French [19, 20], Spanish [21, 22], Thai [23], Japanese [24], Dutch [25, 26], and Cebuano and Wary, two regional languages in the Philippines [27]. Results from these studies dovetail with the findings from Nguyen et al., [12] and suggest that the questionnaire possesses good to excellent internal consistency, and a clear unidimensional factor structure across the different translations [2, 10, 12, 19, 22, 25–28]. In all of the beforementioned validation studies, the questionnaire has been administrated primarily by paper, sent by mail [25, 26], or filled out on-site [2, 10, 12, 21, 22, 29]. Interview has also been used, either on-site [27] or over the telephone [19]. In addition, a validation of a slightly modified version, specifically adapted to measure satisfaction with web interventions, suggests that it might also be suitable for digital use [17]. However, despite its frequent use, a digital version of the CSQ-8 has never been validated. There are often a priori assumptions about the equivalency of digital questionnaires and their pen and paper counterparts, but a systematic review conducted by Alfonsson et al., found that interformat reliability varied from *r* = .35 to *r* = .99 [30]. They proposed several reasons for the disparity: difference in the context where the questionnaire is filled out (e.g., at home instead of on-site), the visual presentation of questions (e.g., one question at a time instead of everything at once), and the level of perceived anonymity. Therefore, equivalency cannot be assumed.

The CSQ-8 has been shown to correlate with treatment outcome, measured both in symptom relief and well-being, and treatment adherence; higher satisfaction is associated with treatment adherence, while dissatisfaction is associated with a higher risk of dropping out [9, 31–33].

The validation of a Norwegian translation of the CSQ-8 is particularly timely. First, over the last few years, it has increasingly been used as a measure of patient satisfaction [14, 15, 34–43]. Second, several registered trials plan to use the questionnaire as an outcome measure [44–48]. Despite this, the psychometric properties of a Norwegian version of the questionnaire have never been investigated. As a digitally administered CSQ-8 is warranted, this study aims to test the psychometric properties of a digital Norwegian version of the CSQ-8.

In line with previous research, we expect the Norwegian digital version of the CSQ-8 to be unidimensional, have good internal consistency, be negatively correlated with change in symptom severity during treatment, and not be correlated with age [2, 12, 49]. We also do not expect sex differences in satisfaction.

## Methods

### Participants and data collection

The data used in this study were collected from 338 patients referred to the outpatient sections of the Nidaros DPS, a community mental health care center in Trondheim, Norway, between March 2020 and September 2021. As part of treatment, every patient received a battery of electronic self-report questionnaires, which were completed a few days before starting treatment, and immediately after treatment termination. Symptom severity was measured both before and after treatment. The CSQ-8 was administered only at treatment termination.

Patients were prompted by a text message inviting them to participate in research and their assessment process. After clicking on the provided link, they were first required to give their consent before answering any of the questionnaires. Both the consent form and the questionnaires were answered on a secure online server provided by the company Checkware AS. Because the results are explicitly used in the treatment assessment process (a therapist can see the self-reported data of her own patients), the degree of anonymity felt by the patients is uncertain.

To be eligible for inclusion in this study, participants had to: be referred to Nidaros DPS for psychological assessment and/or treatment in the given period, provide informed consent for their participation, and be at least 18 years old. Second, patients that did not report symptom severity either before or following treatment termination, or satisfaction at treatment termination were excluded. All other patients were included. The final sample consisted of 338 patients, 66% female, with a mean age of 29.97 years.

This study was approved by the Regional Committee for Medical Research and Ethics in Norway (REK 2019/31,836) and the Norwegian Centre for Research Data (2019/605,327). Every participant was informed that participation was voluntary and that they could withdraw at any time without it affecting any other aspects of their treatment or resulting in any future consequences.

## Measures

### The client satisfaction questionnaire 8

A digital Norwegian version of the CSQ-8 was administrated to the sample to assess overall satisfaction with services in secondary mental health care. The questionnaire consists of eight items, measured on a scale ranging from one to four, with a possible score ranging from eight to 32 [2, 12]. Higher scores indicate higher satisfaction. Two additional open questions to be answered in free text were added; the first asked if the patient had any additional comments, the second asked if they had any suggestions for service improvement.

### The patient health questionnaire 4

To measure change in symptoms, the Patient Health Questionnaire 4 **(**PHQ-4) [50] was used. This is a four-item questionnaire measuring symptoms of depression and anxiety derived from The Patient Health Questionnaire nine items [51] and The Generalized Anxiety Disorder Scale seven items [52]. The first two questions assess symptoms of depression, while the last two assess symptoms of anxiety. All questions are preceded by “over the last two weeks, how often have you been bothered by the following problems?”. Possible answers are presented on a four-point scale ranging from “not at all” to “nearly every day”, scored 0 through 3, making a possible score range of 0–12 [50]. A higher score indicates higher symptom severity. Symptom change was calculated by subtracting the symptom severity score before the start of treatment from the score at treatment termination.

## Statistical analyses

Mean and standard deviation were calculated for the total score of the CSQ-8 and individual items. Descriptive statistics of the sample and Spearman rank-order correlation coefficients were calculated to explore the relationship between age and satisfaction, and the relationship between change in symptoms and service satisfaction. A Mann-Whitney U-test was conducted to test if there was a statistical difference in satisfaction scores between the sexes. Sex was treated as a binary variable, and the one person not specifying their sex was omitted from this analysis.

Score distribution analysis was performed in terms of range, kurtosis, and skew. Floor and ceiling effects were also examined, by calculating the percentage of scores at minimum or maximum values. Terwee et al., [53] have suggested a cut-off of 15% to indicate ceiling or floor effects in a measure.

The inter-item correlation coefficient for each item and Cronbach’s *α* were used to examine internal consistency. The factor structure was evaluated with exploratory factor analysis and principal component analysis, after computing the Kaiser-Meyer-Olkin measure of sampling adequacy and Bartlett’s test of sphericity. Considerations regarding sample size were guided by the Consensus-based Standards for the Selection of Health Measurement Instruments-COSMIN [54]. For factor analysis, the COSMIN recommends a sample size of 7 times the number of items, and > 100 is recommended.

**Fig. 1:**
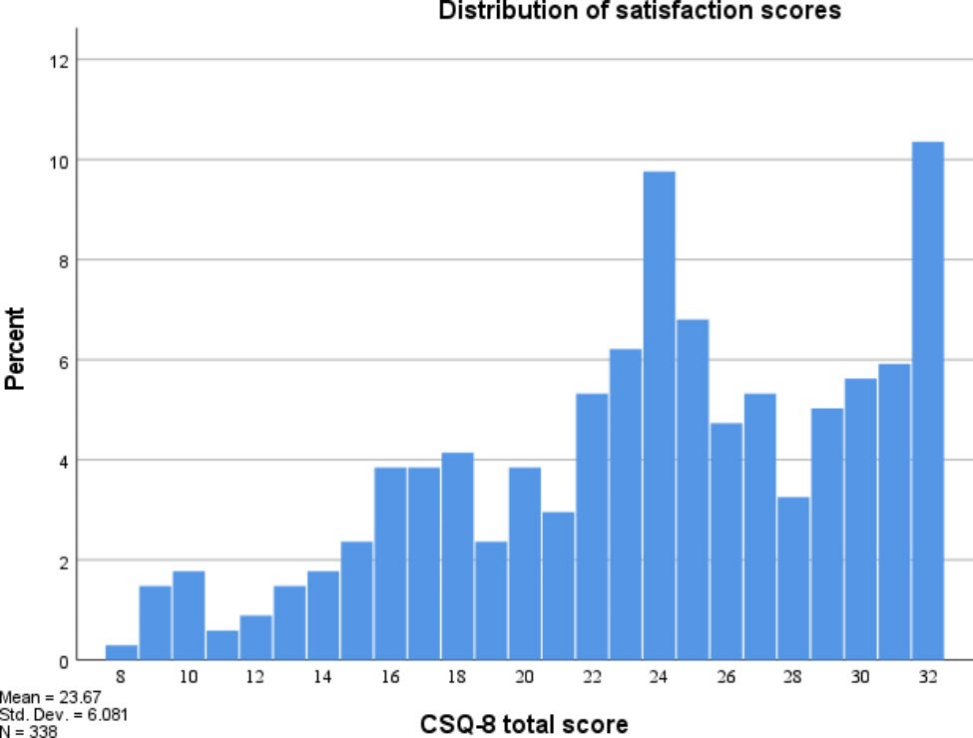
Distribution of Satisfaction scores

No imputations were done, and missing results were excluded pairwise, thus counted as a zero in calculating the total score. All analyses were executed using version 27 of IBM SPSS Statistics.

## Results

### Descriptive statistics of satisfaction scores

Frequencies of different scores for individual items are presented in Table 1. Missing data were less than the recommended cut-off of 5% on all variables [55], with item 8 having the most missing values (2.1%).

**Table 1:** Frequency of scores for each individual item

	Frequency distribution (%)					
Answer	1	2	3	4	Unanswered items (%)	*n*
Item 1: Rating quality of service	18 (5.3)	78 (23.0)	134 (39.6)	108 (32.0)	0 (0)	338
Item 2: Kind of service wanted	15 (4.4)	74 (21.9)	162 (47.9)	87 (25.7)	0 (0)	338
Item 3: Met needs	39 (11.6)	82 (24.3)	143 (42.4)	73 (21.7)	1 (0.3)	337
Item 4: Recommend to a friend	14 (4.2)	30 (8.9)	165 (49.0)	128 (38.0)	1 (0.3)	337
Item 5: Amount of help	34 (10.1)	84 (25.1)	120 (35.8)	97 (29.0)	3 (0.9)	335
Item 6: Improvement in self-efficacy	10 (3.0)	84 (25.1)	123 (36.8)	117 (35.0)	4 (1.2)	334
Item 7: Overall satisfaction	21 (6.3)	76 (22.8)	131 (39.2)	106 (31.7)	4 (1.2)	334
Item 8: Come back	10 (3.0)	55 (16.6)	147 (44.4)	119 (36.0)	7 (2.1)	331

Means and standard deviations for each item are presented in Table 2. Figure 1 shows the distribution of CSQ-8 scores for the sample (*M* = 23.67, SD = 6.08, Median = 24.00). The scores were not normally distributed, indicated by a significant Shapiro-Wilk test (*p* < .001), and negatively skewed (Skewness = − 0.50, *SE =* 0.13; Kurtosis = − 0.50, *SE* = 0.26). The maximum score (32) was the most common (10.4%). Of the 338 patients in the sample, 112 (33%) answered one or both open questions.

**Table 2:** Descriptive statistics and factor loadings of individual items of the Norwegian CSQ-8

Items	M	SE	Factor 1 loadings
Item 1. Rating quality of service	2.98	0.88	0.866
Item 2. Kind of service wanted	2.95	0.80	0.884
Item 3. Met needs	2.74	0.93	0.870
Item 4. Recommend to a friend	3.21	0.77	0.842
Item 5. Amount of help	2.84	0.96	0.833
Item 6. Improvement in self-efficacy	3.04	0.85	0.833
Item 7. Overall satisfaction	2.96	0.89	0.937
Item 8. Come back	3.13	0.79	0.814
Total score	23.67	6.08	

### Correlations with, and sex difference in, satisfaction

Information about age and sex are presented in Table 3. A Spearman’s rank correlation was computed to assess the relationship between satisfaction and age, and satisfaction and symptom change during treatment. A statistically significant, but small-to-negligible correlation was found between age and satisfaction, *r* (335) = 0.13, *p* = .021. A moderate negative correlation was found between satisfaction and change in symptoms (*r* (321) = − 0.355, *p* < .001) in the data. The Mann-Whitney U test found no statistical difference in satisfaction scores between men (*Mdn* = 25) and women (*Mdn* = 24), *U(N*_*men*_ = 113, *N*_*women*_ = 224) = 36469.50, *z* = -1, *p* =. 100.

**Table 3:** Demographic information, symptom change, age, and its correlations with satisfaction scores

		CSQ-8 total score			
		*n*	%	*r* (Mdn)	*p*
Age	Mean (SD)	29.97(10.84)		0.126	0.021*
Sex		337			
	Female	224	66.3	(24)	
	Male	113	33.4	(25)	
	Not specified	1	0.3		
Symptom change (PHQ-4)		323		− 0.355	< 0.001**

*Note.* CSQ-8 = Client Satisfaction Questionnaire 8; PHQ-4 = Patient Health Questionnaire-4. Symptom change was calculated by subtracting PHQ-4 scores before starting treatment from the scores at treatment termination

### Factor structure

**Fig. 2:**
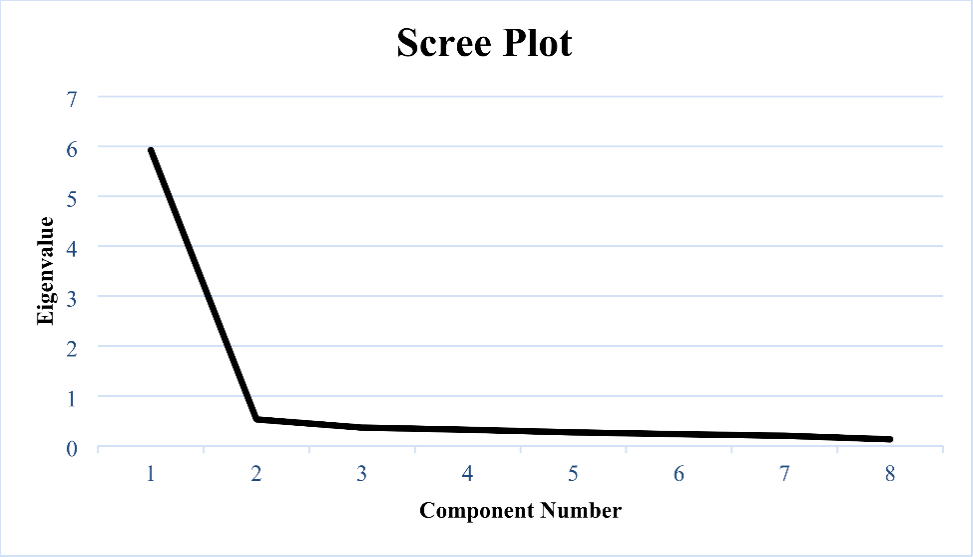
Scree plot of the digital Norwegian CSQ-8

The Bartlett’s Test of Sphericity was significant at the *p* < .001 level, and the Kaiser-Meyer-Olkin Measure was 0.943. Factors were extracted by Kaiser’s criterion. One factor showed an Eigenvalue exceeding 1, explaining 74.1% of the variance (Eigenvalue = 5.93). A scree plot for the data is provided in Fig. 2. All factor loadings exceeded 0.80. An overview of factor loadings is presented in Table 2.

**Table 4:** Internal reliability of the Norwegian CSQ-8 (N = 338)

	Cronbach’s alpha = 0.95	
Item	Corrected Item-Total Correlation	Cronbach’s Alpha if Item Deleted
1. Quality of service	0.82	0.94
2. Kind of service wanted	0.84	0.94
3. Met needs	0.82	0.94
4. Recommend to a friend	0.79	0.94
5. Amount of help	0.78	0.95
6. Improvement in self-efficacy	0.79	0.94
7. Overall satisfaction	0.91	0.94
8. Come back	0.76	0.95
Suitability for factor analysis		
	Kaiser-Meyer-Olkin Value = 0.94	
	Bartlett’s Test of Sphericity: *p < .001*	

### Internal reliability

The CSQ-8 showed very high internal consistency with alpha values of 0.95, with no higher scores if any of the items were to be deleted, and all items showed a corrected Item-total correlation higher than the recommended cut-off of 0.70 [54]. All values are presented in Table 4.

## Discussion

This study aimed to test, for the first time, the psychometric properties of a digital version of the CSQ-8, a satisfaction measure widely used worldwide, which in recent years has seen increased use in Norway.

The Norwegian version appears to have a solid unidimensional structure and very high internal consistency. The amount of variance explained by the single factor is comparable to the normative English American sample (74.1% compared to 75%) [2, 12]. In other validation studies, the total variance explained has ranged from 48.8% (Eigenvalue = 3.89) in the Waray translation [27] to 65.5% (Eigenvalue = 5.24) in the Dutch translation [26]. A shorter version of the CSQ-8, the CSQ-3, consisting of item 3, 7, and 8, has been suggested after analysing the factor loadings that emerged out of the normative sample [12]. In this study, item 2, 3, and 7 had the highest factor loadings, with item 7 having the highest. All factor loadings are presented in Table 2. An alpha value of 0.95 is higher than other alpha values in the literature, which range from 0.80 [22] to 0.94 [55], and 0.87, the value found in the normative English sample [12].

As stated in the introduction, we hypothesized a priori that satisfaction scores would be correlated with treatment characteristics and not client variables. Our results corroborate this hypothesis as we observed a small-to-moderate negative correlation with change in symptoms and no statistical difference in satisfaction between sexes. However, contrary to our hypothesis, we found a weak-to-negligible relationship between age and satisfaction. This mirrors findings from previous validation studies [2, 28, 29, 33, 49]. It is worth noting that there are clinical contexts where we might expect patient characteristics (gender, age differences or other characteristics) to be associated with satisfaction, and contexts where symptom change may not be associated with satisfaction. These contexts include palliative care or the support of people with chronic or degenerative diseases. In turn, patient characteristics may be relevant in treatment settings where one population is in a minority, in populations with heightened stigma, or in more specialized treatment approaches of psychiatric problems which has a different expression in different genders, such as ADHD, autism spectrum disorder, or eating disorders.

As found by earlier studies, the satisfaction scores were in general high, with a mean of *M* = 23.67, and negatively skewed. Ceiling effects is a common problem with satisfaction measures. In the validation study of the Castilian Spanish version, 20% of participants obtained the maximum score of 32 [22]; in the validation study of the Dutch version, 13% obtained the maximum score [26]; Kelly et al., [28] validated the English version in a substance abuse population, here, 20% obtained a score range of 31–32. Although the maximum score of 32 was the most common (10.4%) in our study and below the proposed cut-off of 15% for ceiling effects [53], the distribution of scores on individual items is concerning (presented in Table 1). This is further discussed below.

It may also be worth noting that item 3 (“To what extent has our service met your needs?”) had the lowest mean in our sample, a tendency seen in other studies as well [10, 12, 22, 28]. Although “overall satisfaction” is a broad term, these results imply that the Norwegian CSQ-8 is a narrow measure of a general sense of satisfaction and may not capture specific domains of patient dissatisfaction that occur in parallel with specific domains of satisfaction.

Even though psychometric equivalency between paper and digital versions cannot be assumed, the CSQ-data derived from the online server performed well and our findings are comparable to the original English version, and other validated translations[2, 10, 12, 19, 22, 25–28]. Our study found that the digital version of the CSQ-8 showed a solid factor structure and very high internal reliability, implying that it is a valid measure of overall satisfaction with mental health care services. However, as a good measure of global, overall satisfaction, the CSQ-8’s ability to capture dissatisfaction over specific domains of the treatment context may be limited. A low score does not reveal much information about the treatment received and gives few details about the actual problem or cause of dissatisfaction. The inclusion of two additional open questions at the end of the questionnaire, answered in free text, may remedy this problem. One-third of the participants answered at least one open question. This implies that people see these questions as an opportunity to communicate any idiosyncratic needs or feedback that the other questions did not cover.

There is uncertainty surrounding the degree patients felt their answers were anonymous. However, the research on the effects of administrating the CSQ-8 anonymously is not conclusive, where some research has found an effect [56] while other research with a much bigger sample (1397 compared to 100), albeit with a modified version of the CSQ, has not [57]. Although the measures of symptom severity are included and explicitly used by the therapists during assessment, the CSQ-8 may be in a special position since its content does not directly address themes relevant to the treatment process, but the process itself. More research is needed, however, before concluding anything about the beliefs of patients answering the CSQ-8. Which people do they believe have access to their answers? What type of feedback do they think the CSQ-8 asks for specifically and in what context will it be used to evaluate and maybe change existing practices?

In addition, in our sample, the scores were negatively skewed with a high percentage of maximum scores across individual items, giving rise to the question of whether or not the questionnaire is a good differentiator between nuances of high satisfaction and the degree of responsiveness to improvements in satisfaction [53]. To deal with ceiling effects in other satisfaction measures, a 5-point instead of a 4-point scale has been suggested [58]. However, a possible explanation for this might be that these observed ceiling effects might be better explained by selection bias, which is further discussed below.

The findings of this study should be seen in light of its limitations. All patients that gave informed consent before treatment initiation received the digital CSQ-8 at treatment termination. However, we cannot rule out the risk of selection bias. For example, patients dropping out may be less inclined to fill out questionnaires at treatment termination and may therefore be underrepresented in our sample, or patients who were more satisfied with their treatment may be more prone to completing the questionnaire. This may also explain the skewness and potential ceiling effects observed in our data. Further research should thus investigate if there are patient or treatment course characteristics that are systematically associated with not answering satisfaction measures. Also, as measuring satisfaction at the end of treatment might be vulnerable to bias, more studies where satisfaction is measured at multiple time points during treatment are needed. This is necessary to further assess the psychometric properties of the Norwegian CSQ-8 by making investigations of test-retest reliability and sensitivity to change possible.

Although mean age was assumed to be representative of the Norwegian outpatient population in community mental health care centers [59], our sample was fairly young (mean age 29.9 years) and generalization to less digitally literate populations should be done with caution (e.g., a geriatric population).

Despite its weaknesses, the strengths of the study should also be highlighted. First, data were collected in a large and appropriate sample of the target population, with an ‘observation to variables’ ratio of 42:1. Second, this study differs from previous validation studies in that it primarily evaluates the digital administration of the CSQ-8 in a clinical setting. Third, adding two open questions to the CSQ-8 is a novelty. This study suggests that a substantial number of patients answer these questions, which in turn can enrich the description of patient experiences if future researchers or clinicians wish to capture more idiosyncratic aspects of patients’ experiences or want more specific feedback on possible improvements in mental health settings. Even though qualitative analysis of open questions is outside the scope of this study, we hope these findings can guide future research which should aim to further explore the utility of such answers alongside aggregated satisfaction scores.

### Conclusion

This study aimed to test the psychometric properties of a digital Norwegian version of the CSQ-8, a measure of patients’ overall satisfaction with treatment. The reported data shows for the first time that the digital Norwegian version of the CSQ-8 demonstrates good psychometric properties, and is comparable to the original English version, and other validated translations. The Norwegian CSQ-8 has excellent internal consistency and a robust unidimensional structure. Potential ceiling effects and sensitivity to change require more research. Specifically, future research designs should focus on measuring patient satisfaction at multiple time points. Despite these limitations, we argue that this study indicates that the digital CSQ-8 is an acceptable and feasible measure of treatment satisfaction in Norwegian outpatient populations in adult mental healthcare.

## Data Availability

The dataset analysed during the current study is not publicly available, but it can be available from the corresponding author on reasonable written request.

## Abbreviations

CSQ: The Client Satisfaction Questionnaire.
CSQ-8: The Client Satisfaction Questionnaire 8-item version.
M: Mean.
PHQ-4: Patient Health Questionnaire 4-item version.
SD: Standard Deviation.
REK: Regional Etisk Kommite. *Translated*:Regional Ethical Committee.
